# Comparison of the Effectiveness of Greater Occipital Nerve Block and Botulinum Toxin Type A in Chronic Migraine: An Exploratory Pilot Study From a Tertiary Care Centre in a Resource-Limited Setting

**DOI:** 10.7759/cureus.105513

**Published:** 2026-03-19

**Authors:** Balveen Singh, Monika Singla, Himanshu Kaushal, Juhika Kaur, Gagandeep Singh

**Affiliations:** 1 Neurology, Mahatma Gandhi Medical College and Hospital, Jaipur, IND; 2 Neurology, Dayanand Medical College and Hospital, Ludhiana, IND; 3 Neurology, Guru Gobind Singh Medical College and Hospital, Faridkot, IND; 4 Neurology, Mahatma Gandhi University of Medical Sciences and Technology, Jaipur, IND; 5 Physical Medicine and Rehabilitation, Mahatma Gandhi Medical College and Hospital, Jaipur, IND

**Keywords:** botulinum toxin a (bonta), chronic migraine, greater occipital nerve block (gonb), headache, preempt protocol, visual analogue scale (vas)

## Abstract

Background

Chronic migraine, defined as ≥15 headache days/month with ≥8 migraine days, is a disabling condition with limited effective treatment options. Greater occipital nerve block (GONB) and botulinum toxin type A (BoNTA) are used in chronic migraine management, yet data from certain regions remain scarce.

Objective

The objective of this study was to demonstrate the safety and efficacy of GONB and BoNTA treatments in patients with chronic migraine.

Methods

This prospective, observational, non-blinded exploratory pilot study included 20 adult patients with chronic migraine at a tertiary hospital in India (March 2020-October 2021). Patients were offered either GONB or BoNTA via a cafeteria approach. GONB was administered under ultrasound guidance using lidocaine and methylprednisolone, while BoNTA was injected following the PREEMPT (Phase III REsearch Evaluating Migraine Prophylaxis Therapy) protocol. Headache diaries tracked frequency, severity (via visual analogue scale (VAS)), and medication use for four weeks post intervention.

Results

Sixteen patients (80%) chose GONB, while four (20%) opted for BoNTA. Mean baseline headache days were 22.63 ± 5.1 (GONB) and 26 ± 4.0 (BoNTA). At four weeks, headache days decreased to 10.81 (GONB) and 12.0 (BoNTA). Mean headache-free days post intervention were 17.19 (GONB) and 15.75 (BoNTA). VAS severity scores improved in both groups, with a marked reduction in high-intensity headache days. No serious adverse events occurred; minor local site pain and transient fatigue were the only reported side effects.

Conclusion

Both GONB and BoNTA significantly reduced headache frequency and severity in chronic migraine patients with favorable short-term safety profiles. GONB, being more accessible and cost-effective, may offer a viable alternative in resource-limited settings. Both GONB and BoNTA are effective and safe in chronic migraine, with GONB offering a cost-effective, practical option for resource-limited settings.

## Introduction

Chronic migraine (CM) is defined as headache occurring >15 days/month for at least three months, of which at least >8 days or more have characteristics of migraine. More prevalent among women, it causes significant disability, contributing to societal and economic burden [1]. Available pharmacological treatments are limited, and commonly used drugs have been investigated only in small studies. Management of CM is further complicated by poor therapeutic adherence due to insufficient efficacy and side effects of therapies [2]. One study reported that <5% of CM patients received appropriate care, which included access, diagnosis, and ongoing treatment [3]. This may be partially attributed to a lack of awareness of the public about available treatment options.

Greater occipital nerve block (GONB) has been employed in acute and prophylactic treatments of migraine for two decades [4]. The rationale for performing GONB in chronic headaches is based on the anatomical connections between the trigeminal and upper cervical sensory fibres at the level of the trigeminal nucleus caudalis. Another injectable therapy is botulinum toxin type A (BoNTA). BoNTA is a neuromuscular blocking agent with a wide range of clinical applications, including conditions characterized by muscle overactivity, autonomic dysfunction, or chronic pain. Its mechanism of action is believed to be due to the inhibition of acetylcholine release from the neuromuscular junction, which leads to a decrease in muscle activity. BoNTA has shown efficacy in the treatment of CM in randomized controlled trials [5,6].

Due to the dearth of safety and efficacy outcomes data regarding these treatment modalities, a current exploratory pilot study was planned with the aim of demonstrating the safety and efficacy of administration of GONB and BoNTA in patients with CM.

## Materials and methods

This was a prospective, observational, non-blinded exploratory pilot study conducted at Dayanand Medical College and Hospital, Ludhiana, Punjab, India, between March 2020 and October 2021. The study was approved by the Research and Ethical Committee of Dayanand Medical College (approval number: BFUHS/2K21p-TH/11463). Informed consent was obtained from all participants.

Study population

**Figure 1:**
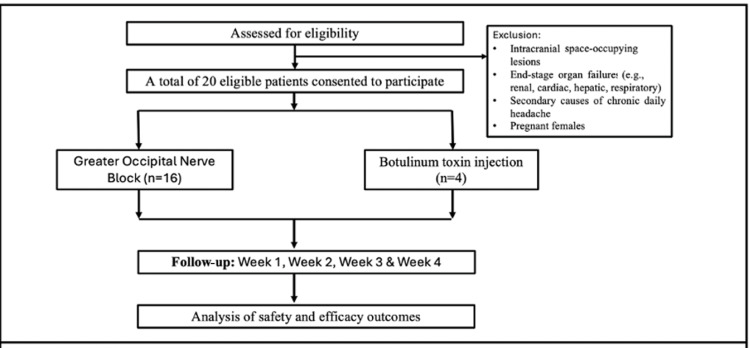
Flow diagram of the assessment of patients, intervention, and outcomes

Patients with CM per International Classification of Headache Disorders, 3rd Edition (ICHD-3) criteria [7], aged 18-80 years, were included; those with intracranial lesions, end-stage organ failure, secondary headache, or pregnancy were excluded. A total of 20 eligible subjects were enrolled (Figure 1). 

Baseline evaluation

Detailed clinical history, neuroimaging (non-contrast computed tomography (NCCT)/magnetic resonance imaging (MRI) brain), and headache diary (baseline four weeks) were recorded. Headache severity was assessed using the visual analog scale (VAS). Details of all concurrent medications, including prescription drugs (with prophylactic therapies) and over-the-counter preparations (including herbal remedies), along with their indications, were recorded, and these treatments were maintained without modification throughout the study duration. 

Interventions

Subjects were explained the procedure in detail and given the option to choose between GONB and BoNTA injection. Patients were explained regarding procedure of both interventions. They were given both interventional procedures as options and were asked to choose between the two by a cafeteria approach. The choice was made based on cost, procedure type, and procedure frequency. A study team member with sufficient experience in injecting GONB and BoNTA administered the injection one time. 

**Figure 2:**
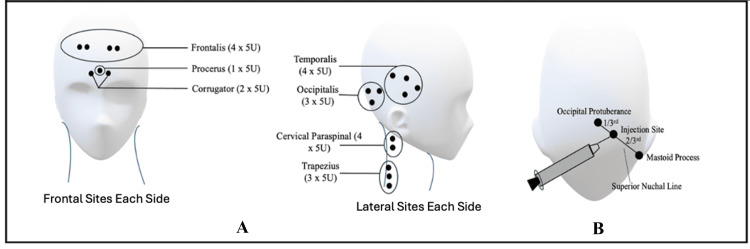
(A) PREEMPT protocol for BoNTA in chronic migraine (Total of 155 units equally distributed among 31 sites over the head) as shown in frontal sites (right and left each) and lateral sites (right and left each); (B) GONB in chronic migraine PREEMPT: Phase III REsearch Evaluating Migraine Prophylaxis Therapy; BoNTA: botulinum toxin type A; GONB: greater occipital nerve block Image credit:Authors; created with Microsoft 365 in PowerPoint (Microsoft Corporation, Redmond, Washington, United States)

For GNOB, an ultrasound-guided occipital nerve block using 3 mL of 2% lidocaine and 80 mg of methylprednisolone was used. The Affiniti 70 Ultrasound system (Koninklijke Philips N.V., Amsterdam, Netherlands) with a high-frequency 6-13 MHz linear transducer was used for localising the greater occipital nerve. Ultrasound probe was placed in a transverse plane at the C2 level, slightly in oblique orientation with the medial end of the probe pointing towards the spinous process of the axis and the lateral end pointing towards the transverse process of the atlas. The greater occipital nerve was identified as a hypoechoic structure between the obliquus capitis inferior muscle and the semispinalis capitis muscle on either side. For BoNTA, the PREEMPT protocol (31 small injections of five units each, total 155 units) (Figure 2) was followed [8,9].

Follow-up

Patients were observed for at least one hour after the intervention. They were asked to fill in a headache diary for four weeks following the injection, recording headache days, severity of headache on a VAS, duration of headache, and medication used at the time of attack. Follow-up in the clinic was done every week for four weeks after the intervention. 

Outcome measures

The primary endpoint was the change in headache days/month. Secondary endpoints were VAS pain score and safety profile (adverse effects, hypersensitivity). At the time of study initiation, we did not anticipate that our observational intervention design would fall under the requirement for prospective registration, and hence, the trial was not registered prior to patient enrolment. Moreover, both treatment modalities are approved for CM in the existing literature, and thus registering this study as a drug trial was not needed. The study just focuses on the comparison between these two therapeutic modalities. 

Statistical analysis

Data were described in terms of range; mean ±standard deviation (SD), median (interquartile range (IQR)), frequencies (number of cases), and relative frequencies (percentages) as appropriate. A probability value (p-value) less than 0.05 was considered statistically significant. All statistical calculations were done using IBM SPSS Statistics for Windows, version 21 (IBM Corp., Armonk, New York, United States).

## Results

Table 1 demonstrates broadly comparable demographic characteristics between groups, with greater variability in headache distribution and trigger patterns among patients treated with GONB. Amongst the 20 subjects, 16 (80%) gave consent for GONB and four (20%) for BoNTA. Among the patients who received BoNTA, all were females, and the mean age was 42.25 years (range 33-55 years). Both groups were comparable in age. Female patients predominated in both cohorts, comprising 100% of the BoNTA group and 81.3% of the GONB group; male patients were present only in the GONB group (18.7%). The mean duration of headache was similar between groups, being 13 years in the BoNTA group and 13.44 years in the GONB group. With respect to headache location, left hemicranial pain was more frequent in the BoNTA group (50%) compared to the GONB group (25%). Right hemicranial headache occurred in 25% of patients in both groups. Holocranial headache was more common in the GONB group (43.75%) than in the BoNTA group (25%). Occipital headache was rare, reported only in one patient (6.25%) in the GONB group. The GONB group showed greater heterogeneity in headache location and triggers, while the BoNTA group had a higher mean number of headache days in the preceding four weeks.

**Table 1:** Baseline demographic and clinical characteristics of the study population BoNTA: botulinum toxin type A; GONB: greater occipital nerve block

Characteristics	BoNTA Group (n = 4)	GONB Group (n = 16)
Age, years (mean)	42.25	42.13
Sex, n (%)		
Female	4 (100%)	13 (81.3%)
Male	0 (0%)	3 (18.7%)
Headache duration (years)	13	13.44
Headache location, n (%)		
Left hemicranial	2 (50%)	4 (25%)
Right hemicranial	1 (25%)	4 (25%)
Holocranial	1 (25%)	7 (43.75%)
Occipital	0 (0%)	1 (6.25%)
Triggers, n (%)		
None	0 (0%)	2 (12.5%)
Fasting	0 (0%)	1 (6.25%)
Sleep deprivation	0 (0%)	1 (6.25%)
Weather change	0 (0%)	1 (6.25%)
Travelling	0 (0%)	1 (6.25%)
Sleep deprivation, fasting	1 (25%)	2 (12.5%)
Stress, sleep deprivation, weather change	0 (0%)	1 (6.25%)
Travelling, fasting	0 (0%)	1 (6.25%)
Travelling, sleep deprivation	1 (25%)	3 (18.75%)
Travelling, sleep deprivation, fasting, stress	1 (25%)	1 (6.25%)
Travelling, sleep deprivation, stress	1	1
Travelling, sleep deprivation, weather change	0	1
Headache days (last four weeks)	26	22.63

Analysis of trigger factors showed greater heterogeneity in the GONB group. No patients in the BoNTA group reported isolated triggers such as fasting, sleep deprivation, weather change, or travelling alone, whereas these were observed individually in small proportions (6.25-12.5%) in the GONB group. Combined triggers were common in both groups, particularly combinations involving travelling and sleep deprivation. The most complex trigger combination (travelling, sleep deprivation, fasting, and stress) was observed in 25% of the BoNTA group and 6.25% of the GONB group.

At baseline, the median number of headache days in the preceding four weeks was higher in the BoNTA group (28 days; IQR: 22-28) compared to the GONB group (20 days; IQR: 20-28). Following intervention, a reduction in median headache days was observed in both groups, with post-injection median headache days of 13.5 (IQR: 7-16.25) in the BoNTA group and 10 (IQR: 6.25-14.75) in the GONB group. The median number of headache-free days after intervention showed a progressive increase over four weeks in the BoNTA group, from two days (IQR: 1.25-3.5) in week 1 to five days (IQR: 5-7.25) by week 4. In the GONB group, median headache-free days were higher in the initial weeks, with six days (IQR: 5.25-7) in week 1 and five days (IQR: 3.25-6) in week 2, followed by a decline to four days (IQR: 3-5) in week 3 and 2.5 days (IQR: 1-4) in week 4.

Prior to intervention, no patients in the BoNTA group reported mild headache severity (VAS 0-4), while 50% each were distributed in the moderate (VAS 4-7) and severe (VAS 7-10) categories. In the GONB group, 12.5% had mild, 37.5% had moderate, and 50% had severe headache severity at baseline. After intervention, median headache severity scores across VAS categories decreased in both groups. In the BoNTA group, median VAS scores were 6.5 (IQR: 5-9.5) for mild, 4 (IQR: 0.5-6.75) for moderate, and 2 (IQR: 0.5-2) for severe headache categories. Corresponding median VAS scores in the GONB group were 5 (IQR: 3.25-9.25), 2 (IQR: 0.5-4.5), and 0 (IQR: 0-2), respectively.

**Figure 3:**
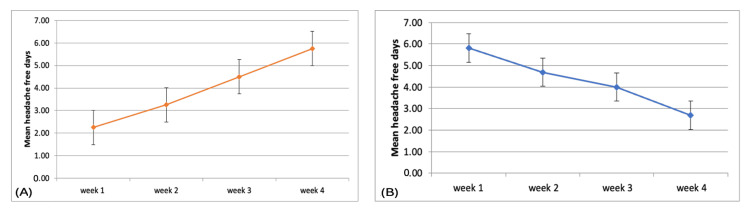
Headache-free days after (A) BoNTA injection and (B) GONB injection BoNTA: botulinum toxin type A; GONB: greater occipital nerve block

Primary efficacy endpoints, secondary efficacy endpoints, and safety outcomes of intervention are summarised in Table 2 and Figure 3. Both interventions resulted in a significant reduction in headache days and severity over four weeks, with GONB showing earlier headache-free days, particularly in week 1, while BoNTA demonstrated a more sustained increase by week 4. Adverse events were mild and comparable between groups, with no serious complications reported. There was a progressive increase in mean headache-free days over four weeks in the BoNTA group, indicating a sustained therapeutic effect, whereas the GONB group showed an early peak benefit in week 1 followed by a gradual decline over subsequent weeks. At baseline, the majority of patients in both groups reported regular analgesic consumption, with higher frequency and quantity of use observed in the botulinum toxin group. Several patients in this group used ≥10-20 tablets per month, and one patient reported consumption of 60-90 rizatriptan tablets per month, suggesting probable medication overuse. In contrast, although all patients in the GONB group were regular analgesic users, their absolute consumption was comparatively lower. Following intervention, a substantial reduction in analgesic use was observed in both groups; however, this reduction was more pronounced in the botulinum toxinBoNTA group, where a marked decrease in both frequency and dependence on analgesics was noted, with 50% of patients becoming completely analgesic-free. In the GONB group, although a reduction in analgesic requirement was evident, complete cessation was less frequent when compared with the botulinum toxin group.

**Table 2:** Results of intervention in both the groups BoNTA: botulinum toxin type A; GONB: greater occipital nerve block; VAS: visual analog scale

Characteristics	BoNTA group (n = 4)	GONB group (n = 16)
Headache days prior to injection in last 4 weeks, mean±SD	26 ± 4.00	22.63 ± 5.18
Headache days after injection, mean±SD	12.25 ± 5.12	10.81 ± 6.32
Headache -free days after intervention, mean±SD		
Week 1	2.25 ± 1.25	5.81 ± 1.42
Week 2	3.25 ± 2.06	4.69 ± 1.88
Week 3	4.50 ± 0.57	4.00 ± 1.93
Week 4	5.75 ± 1.50	2.69 ± 1.66
Mean headache severity prior to injection (VAS), n (%)		
0-4	0 (0%)	2 (12.5%)
4-7	2 (50%)	6 (37.5%)
7-10	2 (50%)	8 (50%)
Mean headache severity after injection (VAS), mean±SD		
Headache days with severity 0-4	7.00 ± 2.44	7.31 ± 6.67
Headache days with severity 4-7	3.75. ± 3.30	2.63 ± 2.18
Headache days with severity 7-10	1.5 ± 1.0	0.88 ± 1.25
Adverse events, n (%)	2 (50%)	7 (43.75%)
Injection site pain	1	4
Abdominal distension	0	0
Injection site paraesthesia	0	0
Fatigue	0	0
Nausea	1	2
Pain (unspecified)	0	1
Vasovagal syncope	0	0

Overall, the onset of BoNTA effect was delayed as compared to GONB, which was almost immediate, early, and excellent. However, over a follow-up period of four weeks, maximum reduction in headache days, or in other words, the maximum number of headache-free days, was more in BoNTA in comparison with GONB (BoNTA 12 days vs. GONB 5.81 days).

## Discussion

Migraine is a common, primary headache disorder characterised by a combination of headache with one or more other neurological, gastrointestinal, and autonomic symptoms. Although highly prevalent and disabling, it is underdiagnosed and under-treated. Despite advances in migraine management, many patients still experience suboptimal relief or side effects with pharmacologic treatments.

This study aimed to evaluate the safety and efficacy of BoNTA and GONB as interventional options for CM. The mean age is similar to the studies by Blumenfeld et al. [10] and Ahmed et al. [11] (Mean age 43 and 45.4 ± 11.7 years, respectively). Vigano et al., however, reported a slightly lower mean age (36.1 ± 7.8 and 32.9 ± 14.5 years, respectively) [12]. Female preponderance (85%) was similar to studies by Blumenfeld et al. [10] and Ahmed et al. [11] (85% and 85.3%, respectively). The mean headache duration was 13±5.75 years in our study, which was slightly more than the duration of 10.6 years reported by Blumenfeld et al. [10] in their study but less than the duration of 20 (10.8-25.3) years observed by Cuadrado et al. [9].

In our study, the number of headache days prior to BoNTA injection in the last four weeks was 26 days, similar to what Blumenfeld et al. observed in their study (22.0 ± 4.8 headache days in the last month before injection) [10]. Of patients who received BoNTA injection, 50% had a headache in the left hemicranial area, whereas 44% of patients who received GONB injection had a holocranial headache. In the study done by Blumenfeld et al., 46.6% of patients complained of headache on both sides of the head [10], while Inan et al. reported that 45.5% of patients had a holocranial headache [13].

In the four weeks preceding the BoNTA injection, patients reported an average of 26 days of headache, which was reduced to 12 headache days after the injection. Similarly, Ahmed et al. found in their study that at baseline, the mean (SD) headache-day frequency was 20.6 ±5.4, which reduced to 7.4±6.6 at administration visit 8 [11]. Blumenfeld et al. also observed that the mean number of headache days reduced to 11.3±7.4 days per 28-day period from 22.0±4.8 days [10]. Young et al. observed that while the baseline mean headache days in the last four weeks were 20.3±4.0, they reduced to 11.8±6.9 post-BoNTA injection [6]. On comparing the GONB group, the patients had an average of 22.63 headache days prior to the injection in the last four weeks, while after the injection, it reduced to 10.81 mean headache days. Inan et al. also reported a reduction in the number of headache-free days from 16.9 ±5.7 pre-treatment to 8.4 ± 5.0 in the second month post-GONB injection [13].

Prior to the BoNTA injection, among the four patients, the mean headache severity in terms of the VAS was in the range 4-7 for two patients, while it was severe (7-10) for two patients. After the BoNTA injection, we compared the number of headache days on the basis of severity. The number of headache days with higher severity was found to be decreasing. The patients had seven mean headache days with severity 0-4, while they had 3.75 mean headache days with severity 4-7 and only 1.5 mean headache days with severity 7-10 on the VAS. Similarly, prior to the GONB injection, among the 16 patients who received it, eight had a mean headache severity in the range of 7-10, while six patients had a severity range of 4-7, and two had a severity range of 0-4. 

In the current study, it was found that the onset of the BoNTA effect was delayed as compared to GONB, which was almost immediate, early, and excellent. However, over a follow-up period of four weeks, the maximum reduction in headache days, or in other words, the maximum number of headache-free days, was greater in BoNTA in comparison with GONB. It has been seen that the onset of the effect of GONB on photophobia and allodynia occurs within 10 minutes of injection, and the reported rate of response is approximately 20% reduction in pain. The reason for this is the sudden inhibition or shutdown of nociceptive impulses from the peripheral receptors in GONB, which gives an almost immediate and faster onset of effect. Moreover, the transmission blockade leads to decreased hyperexcitability of the trigeminovascular system. The effect of BoNTA is proposed to be mediated through the inhibition of synaptic transmission of neurotransmitters in the peripheral nociceptive pathway. It is further mediated by inhibition of SNARE (Soluble -ethylmaleimide-sensitive factor Attachment protein REceptor)-mediated vesicular transport to the presynaptic membrane. This effect takes time for onset, but the effect is more sustained and prolonged [14]. 

No serious adverse events were reported in either group. Injection site pain lasting less than 24 hours was noted in one patient, and one patient observed fatigue for five days post intervention in the BoNTA group. Ahmed et al. discovered in their study that only 18.3% of participants reported adverse effects [11]. Out of these, 7.1% reported mild to moderate adverse effects, while 3.8% reported severe adverse effects. Adverse drug reaction (ADR) in >2% of patients were ptosis (5.4%), neck pain (2.8%), and musculoskeletal stiffness (2.7%). Blumenfeld et al. reported a few adverse effects in around 18.3% of the total patients enrolled [10]. These included ptosis and rash, at 0.4% each. In their study, Young et al. discovered that adverse effects occurred in 14.5% of patients with daily headaches [6]. Injection site pain lasting <1 hour was noted in four patients following the injection in the GONB group. There was no statistically significant difference in adverse events between the BoNTA and GONB groups in the current study. 

Moreover, regarding the concurrent use of medications, the greater reduction in post-intervention analgesic use observed in the BoNTA group, including a higher proportion of patients achieving complete discontinuation, suggests a more sustained preventive effect and a potential role in mitigating medication overuse compared with GONB, although these findings should be interpreted cautiously given the small and imbalanced sample sizes.

**Figure 4:**
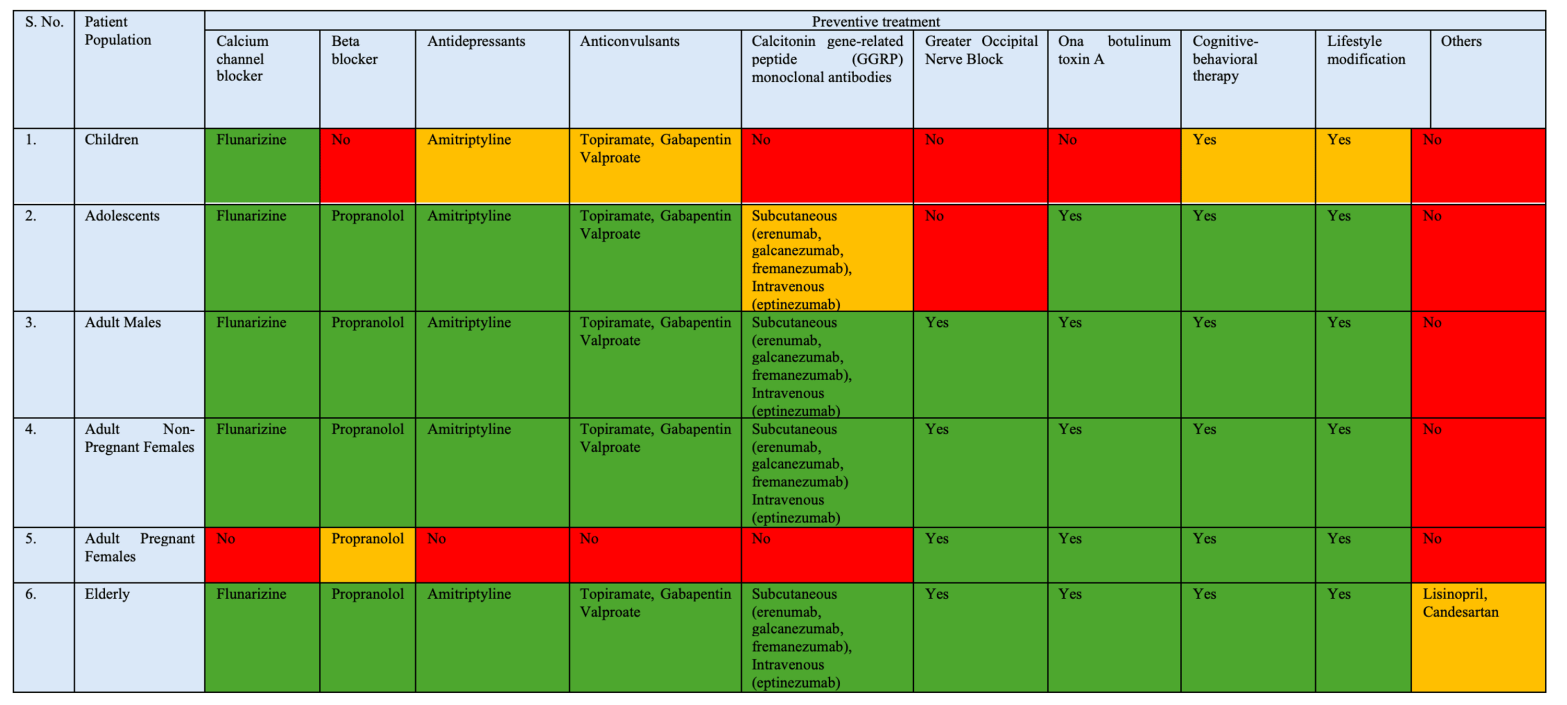
Preventive treatment options for chronic migraine across children, adolescents, adults, pregnant females, and the elderly Red = Not indicated, No Evidence Available; Yellow = Can be Used; Green = Indicated or Evidence Available. This has been compiled and created by the authors after review of published literature [15–17].

**Figure 5:**
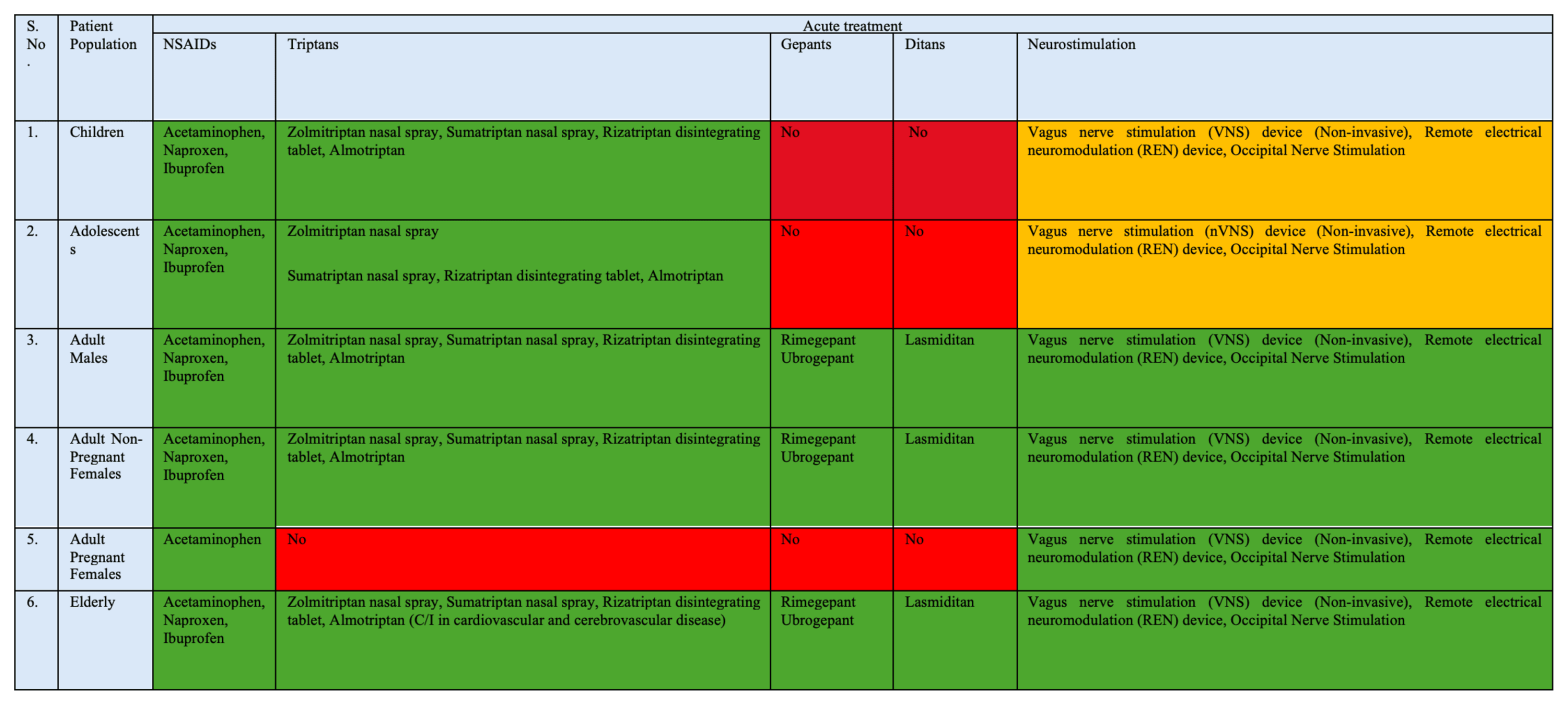
Acute treatment options for chronic migraine across children, adolescents, adults, pregnant women, and the elderly Red = Not indicated, No Evidence Available; Yellow = Can be Used; Green = Indicated or Evidence Available. NSAIDs: nonsteroidal anti-inflammatory drugs This has been compiled and created by the authors after review of published literature [15–17].

Figure 4 and Figure 5, compiled from literature [15-17], show age and population-specific migraine management algorithms, outlining preventive and acute treatment options, respectively, across children, adolescents, adults, pregnant females, and the elderly. They highlight the graded use of pharmacological therapies, newer targeted agents, and neuromodulation, with clear distinctions based on safety, efficacy, and contraindications in each subgroup that act as a simple guide for physicians to choose treatment for chronic migraine. A color-coded scheme has been used to indicate suitability and evidence, where green denotes indicated or evidence-supported options, yellow denotes therapies that may be used with caution or limited evidence, and red denotes therapies that are not indicated or lack evidence.

Figure 4 summarizes preventive treatment options for CM across different patient populations. Preventive therapies are categorized into pharmacological (calcium channel blockers, beta-blockers, antidepressants, anticonvulsants, calcitonin gene-related peptide (CGRP) monoclonal antibodies) and non-pharmacological/interventional modalities (greater occipital nerve block, onabotulinum toxin A, cognitive-behavioral therapy, lifestyle modification, and other agents). Flunarizine, propranolol, amitriptyline, and anticonvulsants such as topiramate, gabapentin, and valproate are broadly applicable across adolescents and adults, while restrictions are evident in children and pregnant females. CGRP monoclonal antibodies and interventional therapies such as greater occipital nerve block and onabotulinum toxin A are mainly supported in adult populations, with limited or no indication in pediatric and pregnant groups. Non-pharmacological strategies, including cognitive-behavioral therapy and lifestyle modification, are consistently applicable across most populations.

On the other hand, Figure 5 outlines acute treatment options for CM across the same patient populations, covering nonsteroidal anti-inflammatory drugs (NSAIDs), triptans, gepants, ditans, and neuromodulation techniques. NSAIDs and selected triptans (particularly nasal formulations and orally disintegrating tablets) are widely indicated in children, adolescents, and adults, with appropriate cautions in the elderly and contraindications in pregnancy. Newer agents such as gepants and ditans are indicated predominantly in adult and elderly populations, while they are not recommended in children, adolescents, or pregnant females due to insufficient evidence or safety concerns. Neuromodulation modalities, including non-invasive vagus nerve stimulation, remote electrical neuromodulation, and occipital nerve stimulation, emerge as broadly applicable options, particularly where pharmacological treatments are limited or contraindicated.

Strengths and limitations

Strengths of the study are that both of the treatments significantly reduced headache frequency. Given the favorable short-term safety profiles, GONB is more accessible and cost-effective when compared with BoNTA.

The limitations inherent in our study significantly constrain the conclusions that can be drawn from our findings. Our study lacked blinding and a control group, be it active or placebo, which raises questions about the validity of the results. Due to the ongoing COVID-19 pandemic, the sample size of subjects in both the BoNTA and GONB groups was relatively small. There was a notable female preponderance in both groups, potentially limiting the generalizability of our results. The cost-effectiveness of treatment with BoNTA was an important consideration for patients, which may have influenced their willingness to participate in the study. Concomitant medication overuse acting as a potential confounding factor is also a limitation. An important limitation of the present study is the skewed enrollment pattern, with a smaller proportion of participants opting for BoNTA therapy compared to GONB. This imbalance likely reflects real-world factors such as treatment cost, accessibility, and patient preference, but it introduces the potential for baseline imbalance and reduced statistical power for between-group comparisons.

Notably, headache frequency data demonstrated a non-normal distribution, necessitating the use of median values with IQRs rather than means to more accurately represent central tendency. When analyzed using medians, baseline headache burden was broadly comparable between groups, with substantial overlap of IQRs for headache days in the four weeks preceding intervention. This suggests that despite a lower mean headache frequency in the GONB group, both cohorts had a similar baseline headache load when assessed using robust, distribution-appropriate measures. Nevertheless, the unequal group sizes remain a methodological limitation and may influence effect estimates. These findings should therefore be interpreted cautiously, and future studies employing randomized allocation with balanced group sizes are required to confirm the comparative efficacy of these interventions. The primary outcome variable's data were partially dependent on patient recall, which could have resulted in recall bias and inflated response rates. In short, future head-to-head comparative randomised controlled trials are needed to compare efficacy and safety in great depth.

## Conclusions

CM remains a major cause of disability, with limited effective treatments. BoNTA and GONB injections offer safe, well-tolerated, and practical alternatives, especially for refractory cases. Their ease of use and growing anatomical rationale support a shift toward peripherally targeted therapies, reducing reliance on daily oral medications. While therapeutic options for CM remain limited, BoNTA and GONB injections may provide a much-needed solution for patients who have not responded to other treatments.
